# Human iPSC-derived macrophages for studying intrinsic and extrinsic factors in cystic fibrosis

**DOI:** 10.70401/EXO.2026.0005

**Published:** 2026-04-10

**Authors:** Daniel Naveed Tavakol, Pamela L. Graney, Meghan R. Pinezich, Connie Chen, Maria Samaritano, Derek Ning, Katherine M. Cunningham, Gordana Vunjak-Novakovic

**Affiliations:** 1 Department of Biomedical Engineering, Columbia University, New York, NY 10032, USA.; 2 Department of Medicine, Division of Cardiology, Columbia University Medical Center, New York, NY 10032, USA.; 3 College of Dental Medicine, Columbia University, New York, NY 10032, USA.

**Keywords:** iPSC, macrophage, cystic fibrosis, inflammation, extracellular matrix

## Abstract

**Background:**

Cystic fibrosis (CF) is a progressive genetic disease characterized by defective ion transport, mucus accumulation, chronic infection, and inflammation that drive airway damage and ultimately end-stage lung failure. Previous studies show that high levels of proteolytic enzymes in the sputum of CF patients correlate with declining lung function, but the related effects on distal lung extracellular matrix (ECM) and immune responses are unclear.

**Methods:**

To address this gap, induced pluripotent stem cell (iPSC) lines from healthy donors and CF patients were differentiated into macrophages, and stimulated with lipopolysaccharide (LPS) to compare their inflammatory responses. Bulk RNA sequencing, functional assays, and secreted protein profiling revealed key differences between healthy and CF-derived macrophages, providing insight into how these cells may contribute to inflammatory responses in CF patients. Further, human lung ECM from distal CF lung tissue was isolated, used to generate ECM biomaterials, and combined with iPSC-derived macrophages from healthy and CF donors *in vitro*. Macrophage phenotype was evaluated through cytokine profiling and RNA sequencing.

**Results:**

CF macrophage inflammation was dysregulated, with elevated baseline IL-8, IL-18, and MCP-1 expression, and a blunted inflammatory response to CF ECM compared to healthy macrophages. By using CF ECM and healthy macrophages, we characterized how healthy cells may be altered in a persistent CF milieu after anticipated CFTR modulator therapy.

**Conclusion:**

These findings reveal altered innate immune behavior in CF and demonstrate the utility of iPSC-derived macrophages for modeling extrinsic immune-ECM interactions in disease.

## 1. Introduction

Cystic fibrosis (CF) is caused by mutations in the cystic fibrosis transmembrane conductance regulator (CFTR) gene and primarily impacts the lungs, intestines, pancreas, and liver. The most severe disease presentation occurs in the lung, where loss of CFTR function causes mucus build-up, chronic infection, severe inflammation, and ultimately end-stage lung disease^[1]^. Historically, treatment of CF focused on either transplantation of donor lungs or symptom mitigation, rather than addressing the underlying disease-causing mutations. In 2019, a new class of combination modulator drugs (elexacaftor/tezacaftor/ivacaftor) was approved by the Food and Drug Administration (FDA) for use in up to 90% of CF patients, primarily by correcting the F508del mutation and potentiating the expression levels of CFTR protein on cell surfaces^[2,3]^. However, the inflamed airway environment persists even in patients receiving modulator therapy^[4,5]^, with the relationship between immune cells and the inflamed extracellular environment in CF remaining poorly understood.

The inflammatory milieu in CF is governed by interactions between immune cell populations, bacteria, and the lung extracellular matrix (ECM). While the majority of immune studies focus on the role of neutrophils in chronic infection and inflammation in CF, recent studies have revealed that macrophages, which comprise 90% of immune cells in steady-state airways, may be significant drivers of lung inflammation. Once thought to be distinctly pro-inflammatory (M1) or pro-resolution (M2), macrophages are now recognized as a highly plastic cell type capable of adopting a range of phenotypes in response to extracellular environmental changes^[4–6]^. In CF, macrophages demonstrate prolonged inflammation, impaired infection response, and compromised ability to clear *Pseudomonas aeruginosa*, a common CF airway infection^[7]^. However, the extent to which this behavior is driven by intrinsic loss of CFTR or by inflammation of the lung extracellular environment remains unclear.

The majority of clinical and pre-clinical studies of CF have focused on disease manifestations in the proximal airways, as bronchiectasis is a hallmark of CF disease progression. However, other studies, including our own work investigating pathological changes to the ECM in CF, have shown that distal lung ECM and its proteins are pathologically altered in end-stage CF^[8]^. Other studies have shown an imbalanced protease/anti-protease composition in CF sputum^[9,10]^. While CFTR modulator drugs rescue CFTR protein in both airway cells and macrophages, clinical studies investigating changes to sputum and circulating inflammatory markers following treatment with CFTR modulators have found no change or only slight decreases in inflammation following treatment^[11–15]^. *In vitro* studies have corroborated this pattern, finding only moderate cytokine changes in cell supernatant in CFTR modulator-treated CF macrophages^[16,17]^. This suggests that macrophage function in CF may be driven by a combination of intrinsic (CFTR loss) and extrinsic (chronic inflammation, ECM changes) factors and emphasizes the need for improved understanding of mechanisms governing lung inflammation. Here, we combined induced pluripotent stem cell (iPSC)-derived cells and ECM biomaterials to study macrophage phenotypes resulting from intrinsic mutations in CF patient cells, as well as macrophage responses to inflammatory stimuli (i.e., lipopolysaccharide, LPS) and pathologically-altered solubilized human CF lung ECM.

## 2. Materials and Methods

### 2.1 iPSC sourcing

All human iPSC lines were obtained through a material transfer agreement. WTC-11 (GM25256, Coriell) was obtained from B. Conklin, Gladstone Institute. BS2 and FA10 iPSC lines were generated by and obtained from the Columbia Stem Cell Core. F508del, W1282X, and G542X iPSC lines were obtained via a material transfer agreement with the Cystic Fibrosis Foundation. The F508del (Class II), G542X (Class I), and W1282X (Class I, nonsense) cell lines were derived from patients with these specific, commonly-associated CFTR mutations.

### 2.2 iPSC culture

Human iPSCs were maintained in mTeSR Plus medium (Stemcell Technologies, 100–0276) on tissue culture–treated plates coated with Matrigel (Corning, 354230) diluted 1:100 in RPMI 1640 (Gibco, A41923–01). Cells were passaged every 3–4 days upon reaching approximately 70% confluency. For passaging, cultures were dissociated using 0.5 mM ethylenediaminetetraacetic acid (EDTA; Thermo Fisher Scientific, 15575) in phosphate-buffered saline (PBS; Corning, 21–040). Following reseeding, cells were maintained for the first 24 hours in mTeSR Plus medium supplemented with 5 μM Y-27632 dihydrochloride (Tocris Bioscience, 1254) to enhance cell survival. All iPSC lines were routinely screened for mycoplasma contamination.

### 2.3 iPSC-macrophage differentiation

iPSCs were replated onto tissue culture plates coated with Matrigel (Corning, 354230; diluted 1:175) under gentle agitation to promote attachment of approximately 8–16 cell clusters per cm^2^. Cells were initially differentiated into hematopoietic stem and progenitor cells using the STEMdiff Hematopoietic Kit (StemCell Technologies, 05310) following the manufacturer’s instructions, up to day 12 of differentiation. On day 12, non-adherent cells were removed, and the adherent hemogenic endothelial cell population was cultured in X-VIVO 15 Serum-free Hematopoietic Cell Medium (Lonza, 02–053Q) supplemented with 1:100 GlutaMAX (Invitrogen, 35050061), 1:100 2-mercaptoethanol, 100 ng/mL recombinant human macrophage colony-stimulating factor (M-CSF; Peprotech, 300–25), and 25 ng/mL recombinant human interleukin-3 (IL-3, Peprotech, 200–03). The hemogenic endothelial cells were maintained for more than eight weeks with weekly medium replacement, discarding suspension cells during the first week. Myeloid and monocytic lineage cells emerging in the suspension fraction were subsequently collected and replated onto 6-well ultra-low attachment plates (Corning) in X-VIVO 15 medium containing 100 ng/mL M-CSF for at least five days to promote macrophage differentiation. CD14+ macrophages were then isolated using CD14 microbeads (Miltenyi Biotec, 130-050-201) according to the manufacturer’s protocol.

### 2.4 Immunofluorescence

Chambered coverslips (Ibidi, 80827) were coated with fetal bovine serum (FBS) for 1 hour prior to cell plating. 24 hours after cell attachment, macrophages were fixed with 4% Paraformaldehyde Solution in PBS (Santa Cruz Biotechnology, Sc-281692) for 20 min at room temperature, permeabilized in 0.25% Triton X-100 (Sigma, X100–100mL) for 20 min at room temperature, and then blocked with 5% bovine serum albumin (BSA; Sigma, A7906–500G) in PBS for 2 hours at room temperature. iM0 cells were then stained with CD68 (1:50, Abcam, ab955) in 1% BSA in PBS overnight at 4 °C. Residual stain was removed by washing with PBS. Samples were then incubated in secondary antibody (1:1,000, Invitrogen, A-21235) and Alexa Fluor 488 Phalloidin (1:1,000, Fisher, A23179) in 1% BSA in PBS for 1 hour at room temperature, then washed with PBS. Finally, samples were mounted using ProLong Glass Antifade Mountant with NucBlue Stain (Fisher, P36983). Images were acquired using a Nikon Ti2 inverted microscope with an AXR resonant spectral scanning confocal unit, then processed in ImageJ.

### 2.5 Flow cytometry

iM0 cells were detached from ultra-low attachment culture dishes by incubation with TrypLE (ThermoFisher, 12605010) for 3 min. For flow cytometry characterization of surface marker expression, cells were centrifuged, resuspended in fluorescence-activated cell sorting (FACS) buffer (2% FBS, 0.5 mM EDTA), and blocked in FcR blocking solution (Miltenyi, 130-059-901) for 15 minutes at 4 °C. Cells were subsequently resuspended in FACS buffer containing AlexaFluor 700-conjugated anti-human CD45 (BioLegend, 304024, 1:50 dilution), Brilliant Violet 420-conjugated anti-human CD11b (BioLegend, 301324, 1:50), and Brilliant Violet 605-conjugated anti-human CD14 (BioLegend, 301834, 1:50). Cells were then stained with propidium iodide (ThermoFisher), and washed twice with FACS buffer. Data were acquired on a Novocyte Penteon flow cytometer at the Columbia Stem Cell Initiative Flow Cytometry core facility. Data analyses were performed using FlowJo software (BD BioSciences).

### 2.6 Phagocytosis assay

iM0 cells were replated onto FBS-coated wells at a density of 100,000 cells/well of a 96-well plate and incubated overnight in X-VIVO 15 media containing 100 ng/mL M-CSF. The cells were subsequently incubated with fresh media containing 20 L/mL fluorescent red latex beads (Sigma, L3030) for 2 hours at 37 °C. Post-incubation, cells were enzymatically detached, centrifuged, washed twice with FACS buffer, and stained with CD45 and CD14 antibodies for flow cytometry analysis, as described above.

### 2.7 Functional efferocytosis assay

iPSCs were collected via 0.5 mM EDTA dissociation for 5 minutes, resuspended in mTeSR Plus, and either stored at 4 °C (control, healthy iPSCs) or transferred under UV light (3 cm distance, 35 J/cm^2^) for 5–10 minutes (apoptotic iPSC group). Post exposure, cells were incubated for one hour at 37 °C. A fraction of these cells were then labeled with an Annexin V-FITC and PI staining kit, according to the manufacturer’s instructions (Abcam). iPSCs from both groups were then resuspended in CFSE for 20 minutes according to the manufacturer’s instructions (Abcam). Cells were then washed in X-VIVO 15 basal macrophage media (Lonza), and cell suspensions were added to wells of iPSC-derived M0 macrophages for one hour at 37 °C. After this, iMacs were washed with basal media, and collected using TrypLE Express (Gibco) for 10 minutes. Cells were washed and stained at 1:100 for BV421-CD11b (BioLegend), washed twice in FACS buffer (2% FBS and 0.5 mM EDTA in 1X PBS), and analyzed on a BioRad ZE5 cytometer (Columbia CSCI Flow Cytometry core).

### 2.8 Functional tumor cell uptake assay

LM2 subclones of MDA-MB-231 breast cancer cells (provided by the Massagué lab at MSKCC) were collected with TrypLE Express dissociation solution for 5 minutes, washed, and incubated with CFSE solution (Abcam) for 20 minutes at 37 °C. Then, half of these cells were exposed to anti-CD47 antibody, with a fraction exposed to FACS buffer. Then, these cells were washed with FACS buffer, and both conditions were exposed to iMacs for two hours at 37 °C. iMacs were collected with TrypLE, washed with FACS buffer, and stained with BV421-CD11b (BioLegend) for 30 minutes at 4 °C. Cells were washed again with FACS buffer, and analyzed on a BioRad ZE5 cytometer (Columbia CSCI Flow Cytometry core).

### 2.9 RNA isolation

Cells were lysed in Buffer RLT from the RNeasy Micro Kit (Qiagen, 74004) and stored at −20 °C until further processing. Upon thawing, total RNA was isolated using the RNeasy Micro Kit following the manufacturer’s protocol. RNA quality and quantity were evaluated using an Agilent Bioanalyzer and a Qubit 2.0 Fluorometer at Columbia University’s Molecular Pathology Core Facility. Samples with an RNA integrity number greater than 8 were deemed suitable for bulk RNA sequencing.

### 2.10 Bulk RNA sequencing

Total RNA samples were submitted to the JP Sulzberger Columbia Genome Center for sequencing. A poly-A pulldown was used to enrich mRNAs from total RNA samples, then a library was constructed using the TruSeq Stranded mRNA Library Prep Kit (Illumina) following the manufacturer’s protocol. Libraries were sequenced to a targeted depth of ~20 M 100bp paired-end reads on a NovaSeq6000. RTA (Illumina) was used for base calling and bcl2fastq2 (version 2.19) was used for converting BCL to fastq format, coupled with adaptor trimming. We conducted pseudoalignment to a kallisto index created from transcriptomes (Ensembl v96, Human: GRCh38.12) using kallisto (0.44.0).

### 2.11 RNA-sequencing analysis

Data processing and analysis were performed in R/Bioconductor^[18]^. Genes which had at least 10 reads in at least 3 samples were considered to be expressed and subject to further analysis. Multidimensional scaling, principal component analysis, and hierarchical clustering were used to check for outliers. Samples were logcpm transformed with a pseudocount of 1. Multidimensional scaling was performed with the plotMDS function in limma^[19]^. Principal component analysis was performed with the plotPCA command in affycoretools. Hierarchical clustering was performed using the heatmap.2 function in the gplots2 package; only the top 1,000 genes are included for clarity and computational tractability. A Euclidean distance metric and complete linkage clustering were used.

Samples were normalized by the Trimmed Mean Method. Differential expression results were analyzed with the Kyoto Encyclopedia of Genes and Genomes (KEGG) and Biological Process Gene Ontology databases, using a significance cutoff of *p* < 0.001 and |log2 fold change| > 0.6. To identify which genes changed in response to LPS treatment differently in control and CF macrophages, we identified genes that were changed in opposite directions upon LPS treatment in control and CF macrophages statistically significantly (FDR 0.05 for both comparisons).

### 2.12 Preparation of ECM biomaterials

Human CF and control lung tissues were decellularized using a combined detergent and enzyme decellularization protocol. Lung tissue was sliced at 2 mm thickness, submerged in sterile deionized water, and placed on a shaker at medium speed for 30 minutes. Tissue slices then underwent four 2-hour wash cycles with CHAPS detergent (8 mM CHAPS, 1M NaCl, and 25 mM EDTA) with alternating rinses with dH_2_O and 2X PBS. Lung tissue sheets were then rinsed in benzonase precondition buffer (50 mM tris-HCl, 0.1 mg/mL bovine serum albumin (BSA), 1mM MgCl_2_) and subjected to enzyme decellularization by washing with benzonase (90 U/mL; Sigma-Aldrich) in benzonase precondition buffer. Decellularized tissue sheets were biopsied for use as acellular scaffolds, or further processed for generation of matrikine cocktails or pre-hydrogel solutions. To prepare the soluble matrix, decellularized tissue was rinsed with PBS, snap frozen in liquid nitrogen, pulverized into a fine powder using mortar and pestle, and dehydrated using a lyophilizer. The resultant dehydrated powder was digested in a pepsin solution (Millipore Sigma) at pH = 2 for 48 hours or until the ECM was fully solubilized. Full characterization of ECM in healthy and CF lung samples is extensively described in our previous work^[8]^.

### 2.13 Histology

Decellularized lung tissues were fixed in cold phosphate-buffered 4% paraformaldehyde for 24 hours, embedded in paraffin, and sectioned at 5 μm thickness. Sections were stained with hematoxylin and eosin (H & E), Masson’s trichrome, and elastic van Gieson’s (EVG) by the histology service in the Department of Molecular Pathology at Columbia University Medical Center and examined via light microscopy.

### 2.14 Immunohistochemistry

Decellularized lung tissues were de-paraffinized, submerged in boiling citrate buffer (pH 6.0) for antigen retrieval for 20 minutes, and blocked with 10% normal donkey serum in phosphate-buffered saline for 2 hours at room temperature. Primary antibodies were added and incubated for 12 hours at 4 °C, or 4 hours at 25 °C. Secondary antibody was diluted 1:500 and incubated for 1 hour at 25 °C. Sections were mounted in mounting medium (Vectashield Mounting Medium; Vector Laboratories) with 4′,6-diamidino-2-phenylindole (DAPI), cover-slipped, and imaged with a Leica DMi8 fluorescence microscope.

### 2.15 Electron microscopy

Scanning electron microscopy (SEM) and transmission electron microscopy (TEM) were performed according to the protocol detailed in previous studies.

### 2.16 PicoGreen DNA quantification

DNA content of native tissue and decellularized ECM was quantified using a DNA quantification assay (Quanti-iT PicoGreen dsDNA Assay; Invitrogen) according to the manufacturer’s instructions. Samples were digested in papain (250 μg mL^−1^) at 60 °C for 4 hours, and mixed with PicoGreen reagent. Fluorescence emission was measured at 520 nm with excitation at 480 nm, and DNA was quantified using a standard curve.

### 2.17 ECM biomaterial matrikine and cytokine profiling

Liquid chromatography mass spectroscopy (LC-MS/MS) was performed according to the protocol detailed in our previous studies to identify peptides present in ECM biomaterials. Cytokine profiles of ECM biomaterials were determined using the Human Cytokine/Chemokine 48-Plex Discovery Assay (Eve Technologies).

### 2.18 Cell-matrix interaction studies

To investigate the relative contributions of CFTR loss versus pathologically altered ECM to inflammation in iPSC-derived macrophages, we cultured iPSC-derived CF (F508del iPSC line) and normal (WTC11) macrophages alone and in combination with CF and control lung ECM. iPSC-derived macrophages were plated at 1 × 10^5^ cells/well in 24-well ultra-low attachment plates (Corning). After 24 hours, ECM induction was performed by adding soluble ECM cocktails from CF and control lung at a concentration of 1 mg/mL in macrophage-support media (X-VIVO15 media with M-CSF). On day 3, tissue culture supernatant was collected and stored at −80 °C for further analysis. RNA lysis buffer was then added directly to the wells and samples were stored at −80 °C.

### 2.19 Statistical analysis

Statistical analysis was performed using GraphPad Prism 9. Outliers were identified using Grubbs’ method. Statistically significant differences were determined using a two-tailed, unpaired t test assuming a Gaussian distribution. For all comparisons, *p* < 0.05 was considered statistically significant (*p < 0.05, **p < 0.01, ***p < 0.001, ****p < 0.0001). All data are represented as mean ± SEM. A sample size of *n* = 3 biological replicates was used throughout this study, defined as iM0 derived from 3 female iPSC lines and 3 male iPSC lines. For each cell line, 2–3 technical replicates were included for all assays.

## 3. Results

**Figure 1.**
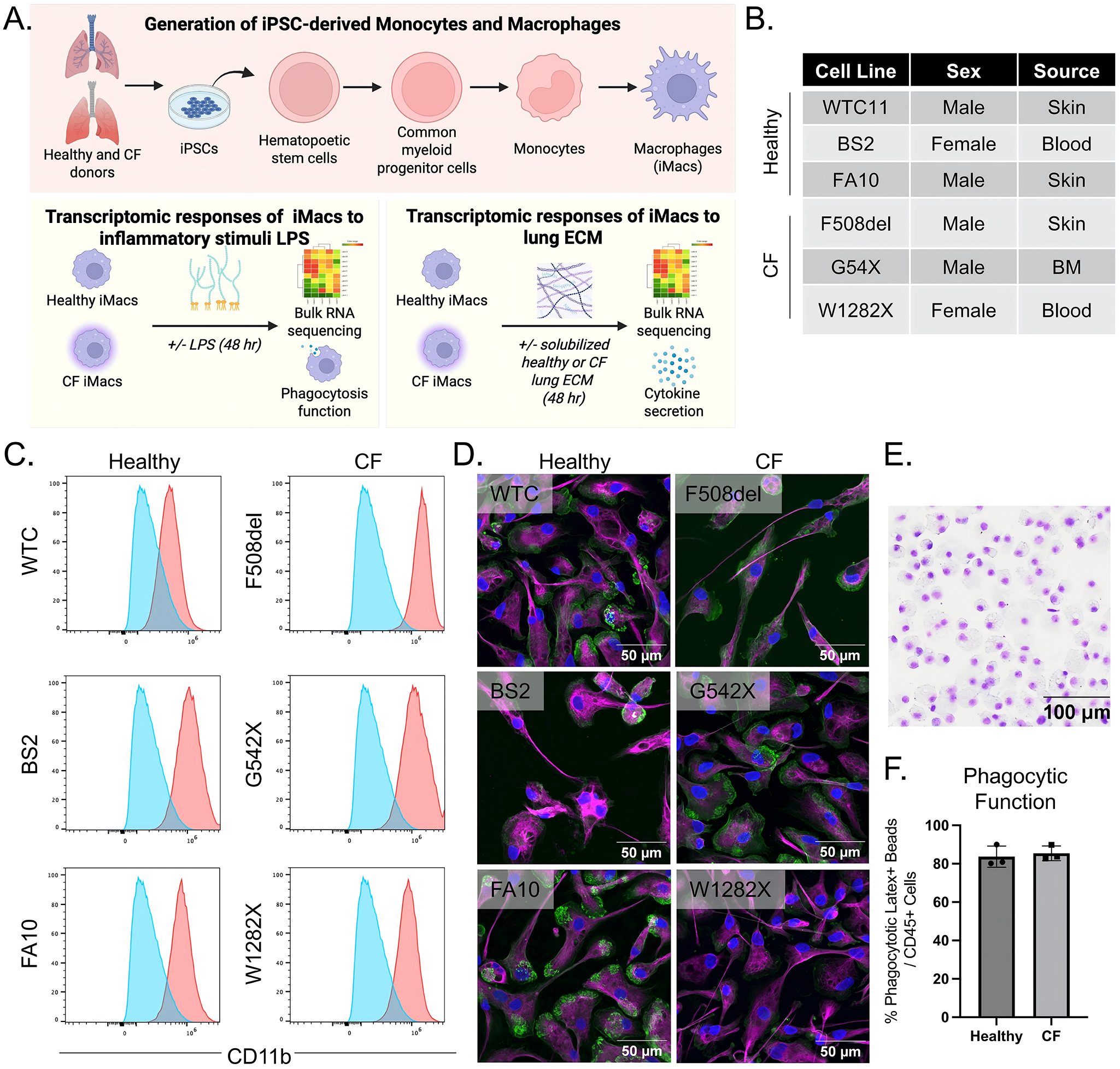
Overview of experimental strategy and derivation of iPSC-derived macrophages (iM0) from healthy and CF donors. (A) Schematic representation of experimental goals; (B) Table of iPSC lines from healthy and CF donors used in the study; (C) CD11b+ flow cytometry profiles of healthy and CF iPSC-derived CD14+ iM0 (red is each donor; blue is the negative control); (D) Immunofluorescent staining of healthy and CF iM0 for CD68 (magenta), phalloidin (green) and nuclei (DAPI), attached on FBS-coated glass cover slips; (E) Example Giemsa-Wright staining showing morphology of cytospun CF iM0; (F) Phagocytic functions of healthy and CF iM0 using phagocytosis of Latex+ microbeads. Created in BioRender.com. CF: cystic fibrosis; DAPI: 4′,6-diamidino-2-phenylindole.

To examine the differences between healthy and CF–associated human macrophages, iPSCs were derived from three healthy donors (WTC11, BS2, FA10) and three donors carrying common CFTR mutations (F508del, G542X, W1282X), and differentiated into macrophages (iM0) following previously published protocols^[20,21]^, as illustrated in Figure 1A,B and Figure S1. iM0 have previously been shown to be similar in their transcriptome to tissue-resident macrophages^[20,22]^. To assess differences between healthy and CF-derived iM0, cells were cultured in serum-free basal medium and stimulated with 100 ng/mL LPS to induce an inflammatory response. Macrophage activation was then evaluated relative to unstimulated iM0 by bulk RNA sequencing, functional assays, and analysis of secreted protein profiles in both healthy and CF donors. These findings were further used to elucidate the contribution of macrophages to inflammatory processes relevant to potential cell replacement therapies for patients with advanced CF-associated matrix remodeling.

### 3.1 Characterization of macrophages from healthy and CF patient iPSC lines

CD14+CD45+ cells were isolated following differentiation from healthy and CF iPSCs. The cells were subsequently characterized by surface marker expression and phagocytic activity to confirm features indicative of macrophages. As shown in Figure 1C, flow cytometry of CD14+CD45+ iM0 detected the presence of canonical myeloid marker CD11b across all lines. Immunofluorescence staining of macrophage marker CD68, phalloidin, and DAPI revealed comparable CD68 expression and cell morphology between healthy and CF patient lines (Figure 1D). Consistent with these findings, there were no significant differences observed in phagocytic function via latex bead uptake between healthy and CF patient line-derived iM0 (Figure 1F). Collectively, these data confirm the successful *in vitro* differentiation of functional iM0 from healthy and CF donor iPSC lines.

### 3.2 LPS stimulation exacerbates CF iM0 phenotypes

**Figure 2.**
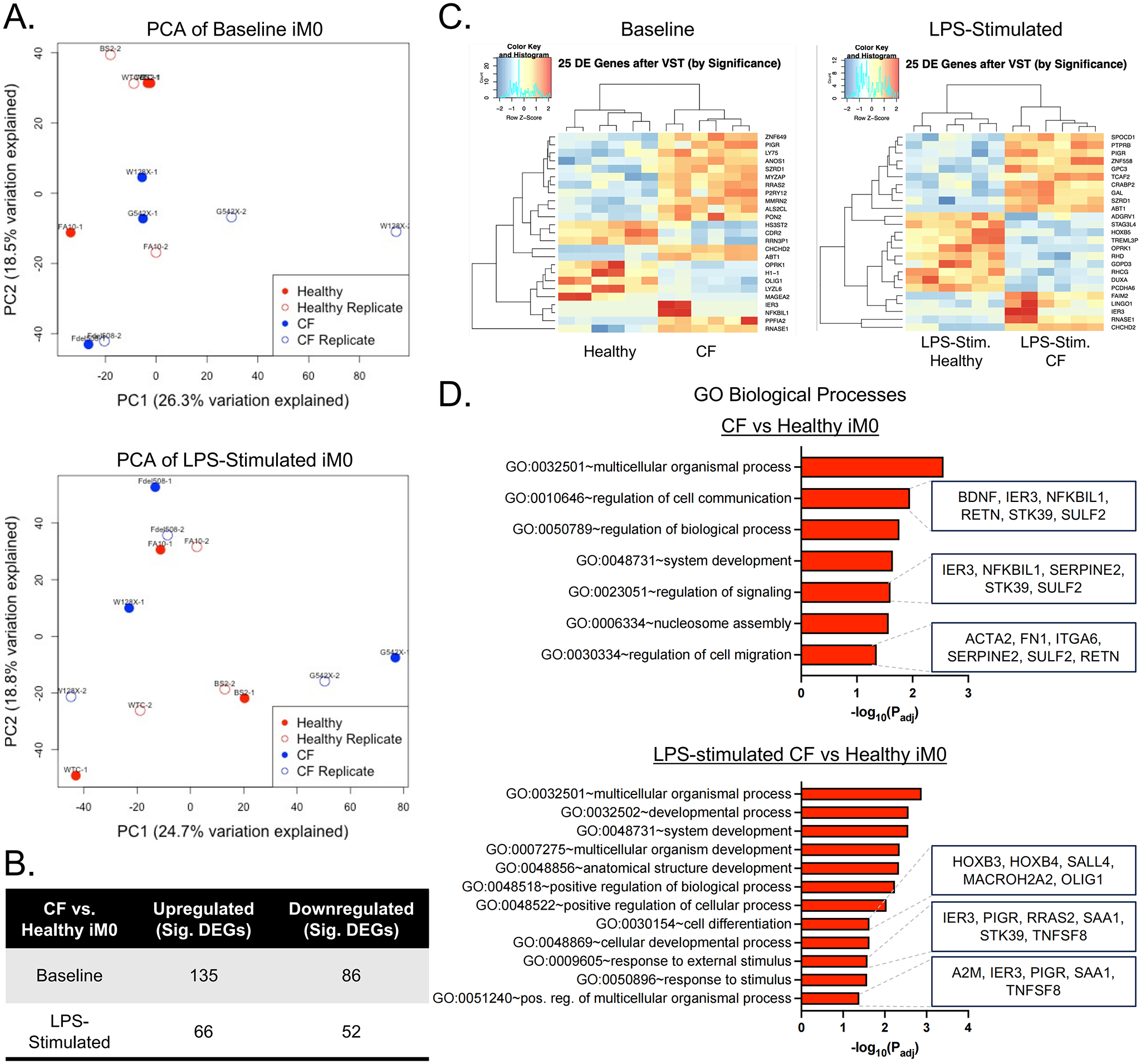
Transcriptomic differences between iM0 derived from healthy (*n* = 3) and CF donors (*n* = 3), with and without LPS stimulation. (A) Principal component analysis of patient-derived iM0 at baseline and in response to LPS showing patient-specific clustering; (B) Comparison of significant differentially expressed genes between groups; (C) Top 25 significant genes for CF iM0 compared to healthy iM0 and GO biological processes pathway analysis; (D) Top 25 significant genes for LPS-treated CF iM0 compared to LPS-treated healthy iM0, with corresponding GO biological processes pathway analysis. Created in BioRender.com. LPS: lipopolysaccharide; GO: gene ontology; CF: cystic fibrosis.

To identify differences in macrophages arising from mutations in the CFTR gene, bulk RNA sequencing was performed on iM0 derived from healthy and CF donors. Notably, principal component analysis of variance-stabilizing transformed data from unstimulated iM0 (baseline) clustered the data according to donor line, irrespective of CFTR status (Figure 2A). Patient-specific differences remained a leading source of variance in the data following LPS stimulation of iM0. Analysis of differentially expressed (DE) genes via DESeq2 identified 221 significant differentially expressed genes (DEGs) in unstimulated CF iM0 compared to healthy iM0 (Figure 2B), and 118 significant DEGs during the inflammatory response to LPS. Hierarchical clustering of the top 25 DEGs separated the data by CFTR status, distinguishing between healthy and CF iM0 in both the absence and presence of inflammatory stimulus (Figure 2C). Further, baseline CF iM0’s had higher gene-level expression of canonical macrophage genes, including *CD14, ITGAM, TLR7*, and *HLA-DRA* (Figure S2).

To investigate the biological processes impacted by iM0 CFTR loss, Gene Ontology (GO) enrichment analysis was performed for DE genes with a log2 fold change > 1 or < −1 and p_adj_ < 0.05 using g:Profiler^[23]^. Genes up- or down-regulated in unstimulated CF iM0 relative to healthy iM0 were enriched in pathways related to the regulation of cell communication and signaling, consistent with the altered cytokine signaling and hyperactivated stress response observed in CF macrophages (Figure 2D)^[24]^. Other notable pathways included “regulation of cell migration” and “nucleosome assembly”, which suggest cytoskeletal and chromatin-level reprogramming of CF iM0 and are consistent with dysregulated chemotaxis in the alveolar space and defective macrophage tissue clearance in CF airways^[25–27]^. In response to LPS, DEGs in CF iM0 were enriched for “cell differentiation”, “response to external stimulus”, and “positive regulation of multicellular organismal process”, reflecting excessive inflammatory signaling and altered differentiation programming of macrophages in response to inflammatory stimuli. These data indicate significant differences in the transcriptomic profiles of healthy and CF iM0 that are exacerbated in the presence of external drivers of inflammation.

### 3.3 Preparation of ECM biomaterials

To study ECM-specific changes to immune cell phenotypes, ECM biomaterials were prepared via detergent and enzyme decellularization of human CF lung tissue. Decellularization resulted in near complete removal of cells and nuclear material as evidenced by lack of DAPI staining on decellularized tissues. DNA quantification was performed on intact native tissue and decellularized biomaterials, revealing > 98% reduction of DNA content following decellularization. Efficacy of decellularization was demonstrated in both lung parenchyma and airways (Figure S3B), where nuclear material was removed leaving ECM, basement membrane, and tissue structure intact.

**Figure 3.**
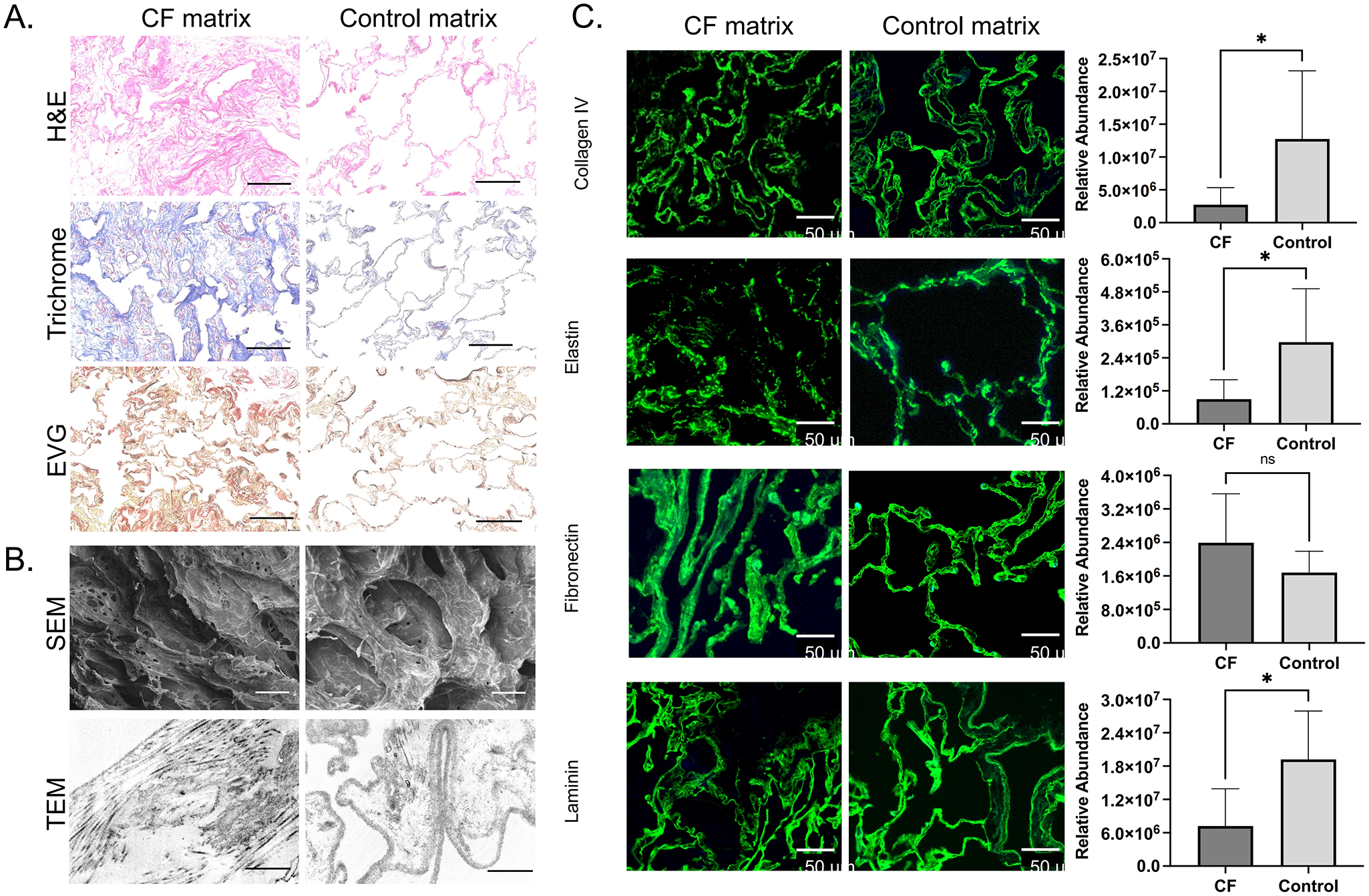
Cystic fibrosis and control lung ECM biomaterials composition and ultrastructure. CF and control (i.e., healthy) lung ECM biomaterials comparison with (A) histology including H & E, Masson’s trichrome (blue, collagens), EVG (black, elastin); (B) electron microscopy including SEM, and TEM; (C) key structural constituents, including Collagen IV, elastin, fibronectin, and laminin (visualized with immunofluorescent imaging and quantified by LC-MS/MS relative abundance). Scale bars: H & E, trichrome, EVG, 100 μm; SEM: 40 μm; TEM, 1 μm. *p < 0.05; **p < 0.01 using a student’s unpaired t-test. Created in BioRender.com. ECM: extracellular matrix; CF: cystic fibrosis; H & E: hematoxylin and eosin; EVG: elastic van Gieson; SEM: scanning electron microscopy; TEM: transmission electron microscopy; LC-MS/MS: liquid chromatography/mass-spectrometry; EVG: elastic van Gieson’s.

Biomaterials were prepared from the CF lung matrix, in the form of decellularized ECM scaffolds and soluble ECM for use as media supplement or plate coating materials (Figure 3A). Following decellularization, lung ECM retained composition and structure similar to fresh fixed lung tissue. Compared with ECM scaffolds derived from control lung tissue, CF scaffolds displayed ECM derangement and disorganization, including fragmented elastin and collagen fibers. On SEM, CF ECM scaffolds demonstrated loss of ultrastructural integrity with alveolar collapse and septal degradation (Figure 3B). Matrix fragmentation was observed in CF scaffolds visualized with TEM (Figure 3B). Key lung ECM components, including collagen IV, elastin, laminin, and fibronectin, were compared in CF and control biomaterials using immunofluorescent staining and protein quantification via LC-MS/MS (Figure 3C). ECM structure was abnormal across all four ECM proteins of interest, and levels of collagen IV, elastin, and laminin were significantly lower in CF compared to control lung biomaterials (*p* < 0.05). Structural properties of CF ECM biomaterials were consistent with observations in CF native tissue reported in our previous studies. Decellularized ECM scaffolds were further processed into soluble matrix cocktails and were compared in composition and structure to native lung tissue and decellularized ECM scaffolds.

Multiplexed quantification of cytokines revealed higher levels of inflammatory cytokines in CF than control lung ECM materials. For example, FGF-2, IL-12P40, G-CSF, Flt-3l, IL-1RA, IL-4, and IL-18 were all significantly elevated in CF ECM cocktails compared to control lung ECM cocktails. The majority of other cytokines measured were elevated in CF materials, though not significantly (Figure S3C).

### 3.4 Co-culture of iM0s in CF ECM

**Figure 4.**
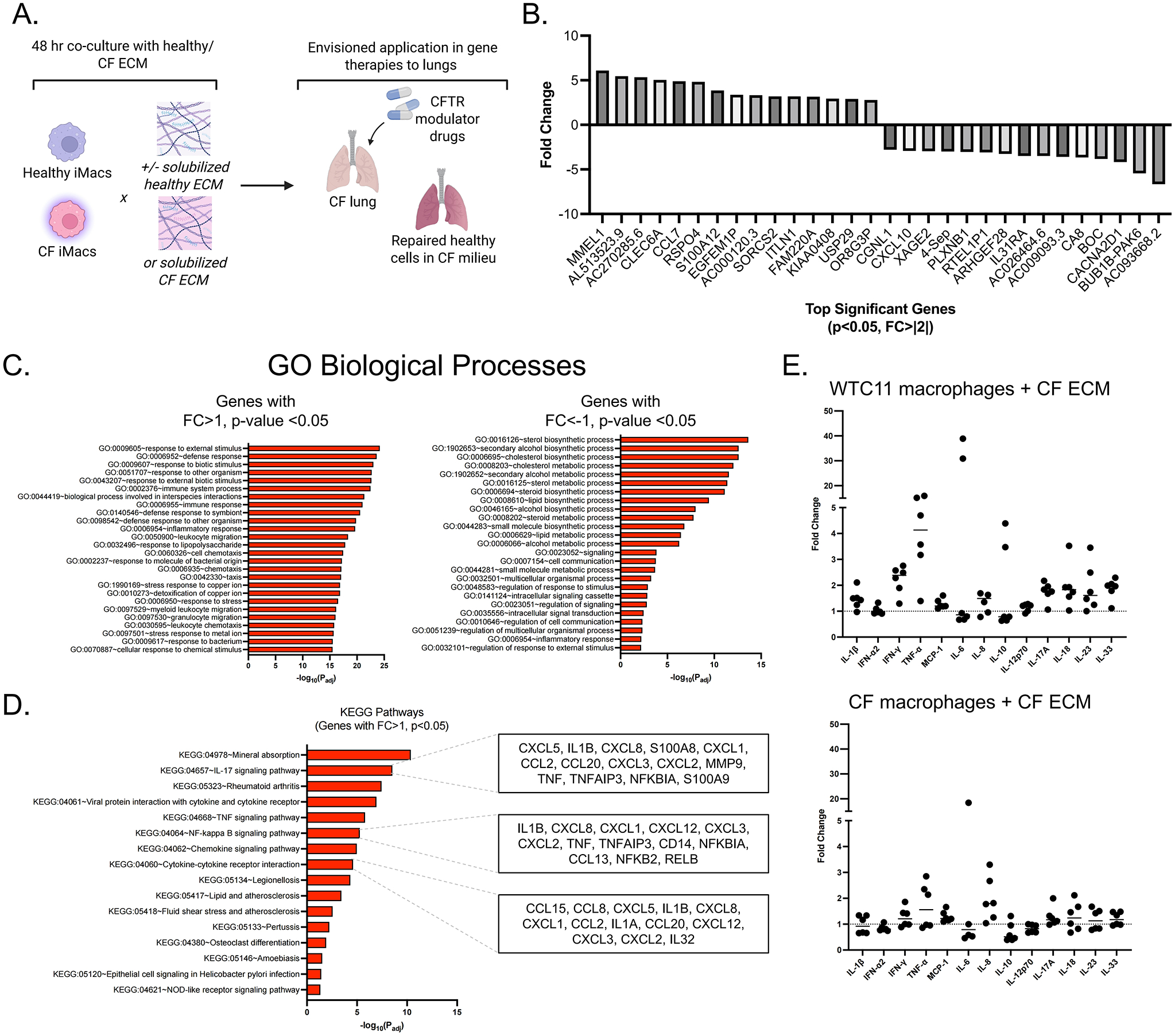
Co-culture of iM0s with healthy or CF ECM materials. (A) Overview of cell and ECM combinations in experimental design, with envisioned applications in understanding how CFTR modulator-rescued cells may behave in a persistently diseased milieu; (B) Top significant (*p* < 0.05, FC |2|) differentially expressed genes in control iM0s exposed to CF ECM for 48 hours; (C) GO biological processes for differentially expressed genes with upregulated (> 1) and downregulated (< −1) fold changes; (D) KEGG pathways associated with upregulated (> 1) differentially expressed genes, with highlighted genes for key pathways; (E) Cytokine fold changes of control (WTC11 line) versus CF iM0s (F508del line) in response to decellularized, and solubilized, CF ECM. Created in BioRender.com. ECM: extracellular matrix; CF: cystic fibrosis; CFTR: cystic fibrosis transmembrane conductance regulator; KEGG: Kyoto Encyclopedia of Genes and Genomes.

To probe intrinsic and extrinsic regulation of macrophage activation, macrophages derived from healthy or CF donor iPSCs were cultured in isolation and in co-culture with solubilized CF or control lung ECM (Figure 4A). Notably, the ECM and iPSC-derived macrophages were not matched. By performing bulk RNA sequencing on healthy iM0s in response to non-CF and CF solubilized ECM, we were able to identify many significant differentially expressed genes in healthy iM0s cultured in the pro-inflammatory CF matrix environment (Figure 4B). In particular, there were significant increases in key matrix remodeling-related genes such as CCL7 and S100A12. By performing GO biological processes pathway analysis, significant pathways associated with upregulated genes (FC > 1, *p* < 0.05) included “response to external stimulus”, “immune response”, and “leukocyte migration”. Downregulated genes (FC < −1, *p* < 0.05) revealed pathways indicative of impaired metabolic functions, including “cholesterol metabolic process”, “lipid biosynthetic process”, and “cell communication”, (Figure 4C). When identifying KEGG pathways associated with upregulated genes (FC > 1, *p* < 0.05), those associated with “IL-17 signaling”, “tumor necrosis factor (TNF) signaling”, “NF-kappa B signaling”, and “cytokine-cytokine receptor interactions” were significant, with associated genes including many CXCL and S100A family genes (Figure 4D).

Cytokine expression of CF versus healthy iM0 was analyzed prior to exposure to each ECM, and MCP-1, IL-8, and IL-18 were found to be significantly elevated in CF macrophages (Figure S4). To delineate the contributions of elevated baseline levels of cytokines observed in CF iM0 prior to ECM exposure from contributions of the ECM itself, all cytokine levels were normalized to baseline values in CF and healthy macrophages. Healthy iM0 demonstrated a stronger inflammatory response (increased fold change relative to baseline) compared to that of CF iM0 exposed to CF ECM (Figure 4E). In particular, healthy iM0 displayed higher fold change values of TNFα and IFN-γ compared to CF macrophages.

## 4. Discussion

The contributions of macrophages to persistent lung inflammation in CF remain unclear. Key questions center on whether dysfunction of macrophages stems from intrinsic defects caused by absent CFTR or from extrinsic cues within the altered CF airway environment. Although CFTR modulators improve lung function and restore CFTR expression in macrophages and other cells, they do not fully correct dysregulated inflammatory pathways or reverse existing tissue damage^[17]^.

Extracellular environmental stimuli that were previously reported as drivers of macrophage phenotype in CF include circulating serum and plasma cytokines, mucus abnormalities, and neutrophil extracellular traps^[15,28,29]^. Decreased phagocytic ability of alveolar macrophages exposed to CF serum or plasma has been reported in multiple studies^[16,17,28]^, and macrophages in the CF airway milieu demonstrate a chronic M1 (pro-inflammatory) state, indicating the importance of the cellular microenvironment in macrophage regulation in CF. Furthermore, excessive inflammation and infection have been shown to persist in the CF lung even after treatment with highly effective CFTR modulator drugs, suggesting that immune and macrophage dysregulation may persist despite CFTR rescue^[12,13]^. Although the classical view of CF identifies the airway as the primary site of disease, CF manifestations that begin in the proximal airways have a cascading effect that includes factors involved in chronic infection and inflammation that impact the distal airways, such as in resident alveolar cells and extracellular proteins^[8,30]^.

In this study, we used CF lung tissue-derived ECM biomaterials and iPSC-derived macrophages to investigate drivers of macrophage activation in CF. Specifically, we generated iPSC-derived macrophages from both healthy and CF donors and characterized them using functional, structural, and transcriptomic assays with and without LPS-stimuli, as a model of lung infection. We then treated a subset of these iM0 with soluble lung ECM isolated from human CF and control lung tissue and determined that iM0 activation is modulated by a combination of both intrinsic (mutation-driven) and extrinsic drivers (extracellular matrix environment).

Bulk RNA sequencing of iM0 from control and CF patients revealed differentially expressed genes indicative of increased inflammatory stress in CF-derived iM0. Pathway analysis indicated increased cell-matrix interactions and humoral immune responses in CF iM0 at baseline, similar to previous studies that showed macrophage regulation of matrix deposition, chronic inflammation, and alveolar fibrosis. Notably, the pathways “regulation of cell communication” (GO:0010646) and “regulation of signaling” (GO:0023051) revealed cytokine- and chemokine-related signatures that align with hallmark inflammatory defects in CF.

Upregulated genes such as *NFKBIL1, IER3, STK39, SULF2, and RETN* point to persistent nuclear factor kappa B (NF-kB) activation and impaired inflammatory resolution, indicating a process of disrupted signaling feedback and macrophage-epithelial cross-talk that characterizes CF airway disease^[31]^. CF macrophages show disrupted motility and adhesion programs, impairing effective pathogen clearance, and differentially expressed genes including *FN1, SERPINE2, ITGA6*, and *SULF2* highlight altered ECM-cytoskeletal interactions that underlie these migratory defects. Prior studies have shown similar dysregulation of adhesion and migratory capabilities in myeloid cells in CF patients, distinguishing between aberrant integrin clustering versus activation in CF^[32]^ and suggesting this phenomenon may be linked to mutations in F508del^[33]^.

CF iM0 cultured under basal conditions also exhibited significantly higher expression of inflammatory cytokines including MCP-1, IL-8, and IL-18, relative to healthy (WTC11) macrophages (Figure S4), suggesting that loss of CFTR alone alters the inflammatory profiles of macrophages. MCP-1 and IL-8 have previously been identified at elevated levels in bronchoalveolar lavage (BAL) fluid of CF patients, as well as patients with other interstitial lung diseases^[26,34,35]^. In particular, MCP-1 BAL levels correlate with alveolar macrophage infiltration, which persists even in the absence of pulmonary infection, while serum IL-8 elevation has been proposed as an early biomarker for inflammation in CF^[36]^.

In response to LPS stimulus, CF macrophages displayed a heightened activation state marked by exaggerated NF-kB/mitogen-activated protein kinases (MAPK) signaling and amplified cytokine responses, similar to the dysregulated inflammatory phenotype observed in patients. Our data suggest that CF macrophages undergo altered lineage and activation programming, consistent with a transcriptionally ‘reprogrammed’ state that limits plasticity and reinforces a maladaptive, pro-inflammatory phenotype aligned with trained immunity in CF. Pathways such as “cell differentiation” and “response to external stimulus” indicate that although CF iM0 may be inflammatory at baseline, upon stimulation with LPS, these cells respond by increasing their pro-remodeling phenotype and likely rely on multicellular interactions with other airway cells, including epithelial cells, fibroblasts, and endothelium, for resolution of triggered inflammation.

We next exposed healthy iM0 to CF ECM to mimic the cell-matrix interactions that occur between CFTR-corrected iM0 and ECM in patients with late-stage disease, such as adult CF patients with significant lung damage who begin treatment with CFTR modulator therapy. We found that healthy iM0 exposed to CF ECM demonstrated an elevated inflammatory profile relative to healthy iM0 at baseline, including expression of chemoattractant pro-inflammatory genes and upregulation of processes associated with increased inflammatory responses, leukocyte migration, and TNF and NF-kB signaling. CF iM0s exposed to CF ECM exhibited slight increases in TNFα and IFN-γ relative to baseline CF iM0, but fold change was lower than that of healthy cells exposed to CF ECM. Taken together, these findings suggest that CF iM0 demonstrate intrinsic differences in cytokine expression and that these differences are exacerbated by the diseased CF lung ECM.

Responses of macrophages to CFTR modulator therapy have been previously investigated due to the known ability of macrophages to express CFTR and mediate inflammation in the lung. While administration of CFTR modulators *in vitro* improved macrophage phagocytosis, increased their ability to kill *Pseudomonas aeruginosa*^[16,37]^, and decreased levels of IL-1β and IL-18^[30]^, the same recovery was not observed in clinical studies, which have shown that chronic infection and inflammation persist despite improved lung function resulting from treatment with CFTR modulators. For example, patients treated with ivacaftor and lumacaftor/ivacaftor had no change in sputum inflammatory markers or circulating inflammatory gene expression 6 months following administration^[11–15]^. Therefore, correction of CFTR is not sufficient to restore macrophage function *in vivo*, possibly due to microenvironmental cues or redundant mechanisms that drive continued expression of inflammatory pathways. When viewed with prior evidence indicating that CFTR restoration *in vitro* is sufficient to recover macrophage inflammatory pathways, our findings indicate that the CF ECM may serve as an additional extrinsic driver of macrophage abnormalities.

While we studied the effect of pathological changes to lung ECM composition on macrophages, our study has several limitations. We investigated the response of macrophages to end-stage CF lung ECM; future studies could employ a similar experimental design to investigate the effects of mucus composition, microbial populations, and tissue stiffness on macrophages and other cell types including neutrophils and mesenchymal stromal cells. Many of these factors could be incorporated in 3D settings to better mimic the physiological microenvironment of the native lung. Capturing the sequential progression of phenotypes that mimic earlier stages of disease could elucidate mechanisms to rescue aberrant inflammatory pathways. Our model system is comprised of human tissue-derived ECM and human iPSC-derived cell populations and could enable patient-specific studies of disease. While we focused on a small sample size (*n* = 3) for this study, future studies could use a larger cohort of iPSC lines, incorporate multiple cell types, and leverage organoid or organ-on-a-chip platforms to account for a wide variety of CF patient mutations and capture complex patient responses to inflammation^[38–43]^. By incorporating fibroblasts, endothelial cells, and parenchymal epithelial cells of the lung, future studies may further elucidate cellular cross-talk that drives matrix deposition and disease progression. Because CFTR plays a key role in lysosomal acidification and reduced bacterial killing in macrophages, and in our study, we did not see this phagocytic reduction in efficiency, a more sensitive phagocytic assay must be used to further confirm the iM0 phenotypes^[44]^. Future studies must also begin to compare how iPSC-derived macrophages compare to bronchoalveolar lavage macrophages in CF patients.

In summary, our findings highlight the use of iPSC-derived immune cells for studying both intrinsic and extrinsic factors implicated in CF disease. By using a combination of control and CF iM0, we demonstrated variability in CF patient cohorts, as well as key genes implicated in iM0 phenotypic responses to intrinsic (LPS) and extrinsic (ECM) inflammatory stress. Further, our model can be applied to evaluate the relationship between CFTR expression and extracellular inflammation in a CF-specific environment. As ongoing inflammatory processes continue in patients receiving CFTR modulator therapy, therapeutics that target dysregulated macrophage patterns could serve as complementary interventions to support tissue repair.

## Supplementary Material

Supplementary Materials

The supplementary material for this article is available at: Supplementary materials.

## Data Availability

Data is available at time of publication from the Gene Expression Omnibus database, or from request to the corresponding author.
