# Supplementary material for: Human iPSC-derived macrophages for studying intrinsic and extrinsic factors in cystic fibrosis
Source: EXO. Author manuscript; Available in PMC 2026 Apr 26. (PMC13110062; doi:10.70401/EXO.2026.0005)
Supplement: Supplementary Materials [file NIHMS2166734-supplement-Supplementary_Materials.pdf]

https://doi.org/10.70401/EXO.2026.0005

Supplementary information

Human iPSC-derived macrophages for studying intrinsic and extrinsic factors in cystic fibrosis

Daniel Naveed Tavakol, Pamela L. Graney, Meghan R. Pinezich, Connie Chen, Maria Samaritano1, Derek Ning, Katherine M. Cunningham, Gordana Vunjak-Novakovic

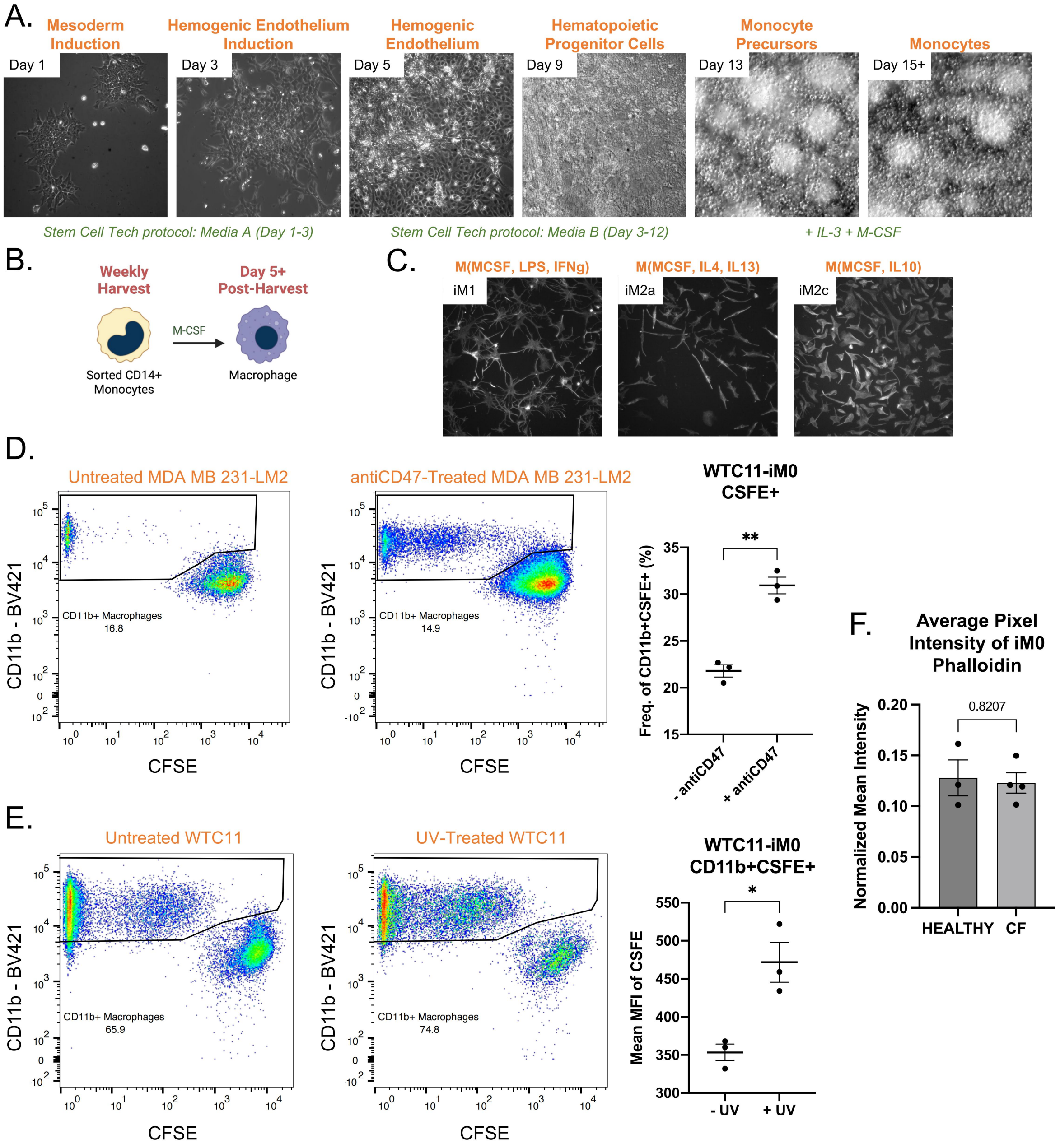

Figure S1. Detailed breakdown of iPSC-to-macrophage differentiation. (A) Differentiation of iPSC-derived monocytes from the Stem Cell Technologies hematopoietic progenitor differentiation kit, with pictures of the step-wise protocol; (B) iPSC-derived monocytes can be harvested weekly, sorted for CD14 expression, and differentiated into macrophages in the presence of MCSF; (C) iPSC-derived macrophages (iM0) can be further stimulated into M1-, M2a-, and M2c-like phenotypes; (D) iM0 phagocytose tumor cells treated with anti-CD47; (E) iM0 efferocytose UV-treated WTC11 iPSCs; (F) Quantification of phalloidin signal intensity of iM0 derived from healthy and CF iPSC, as shown in Figure 1.

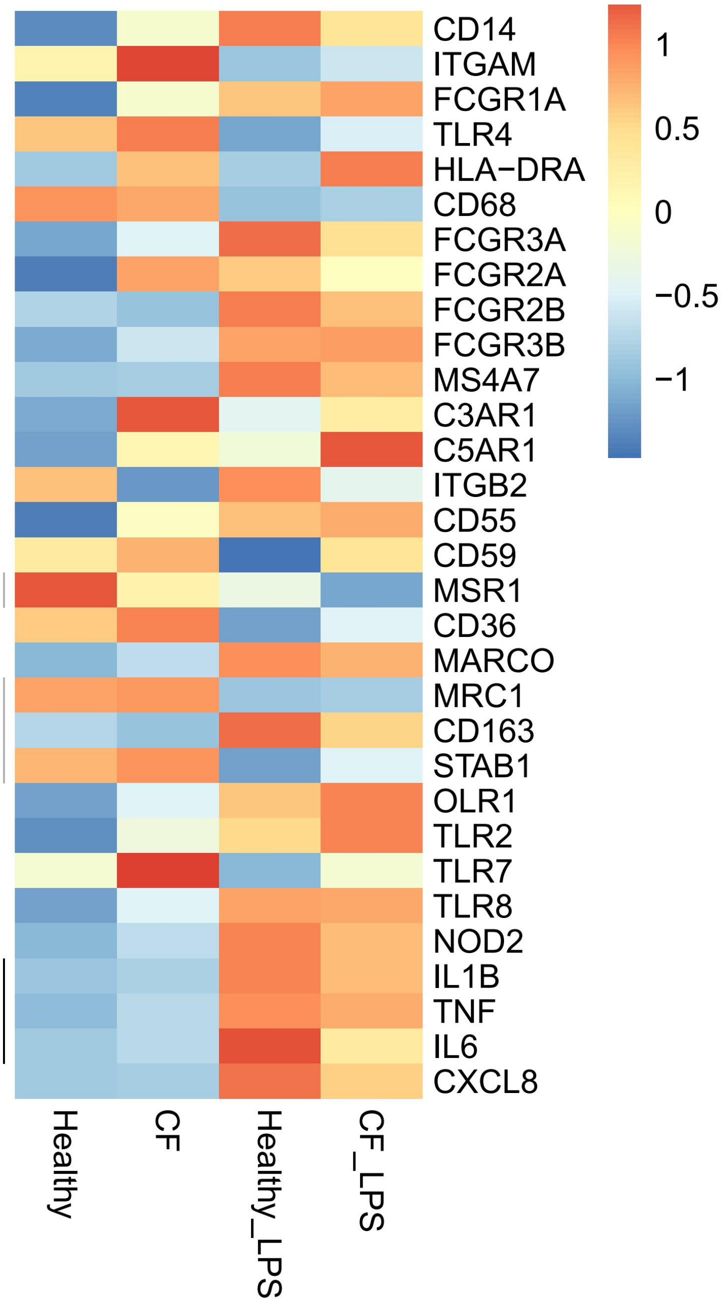

Figure S2. Heatmap of canonical macrophage genes in iM0 at baseline and after LPS stimulation from bulk RNA sequencing data.

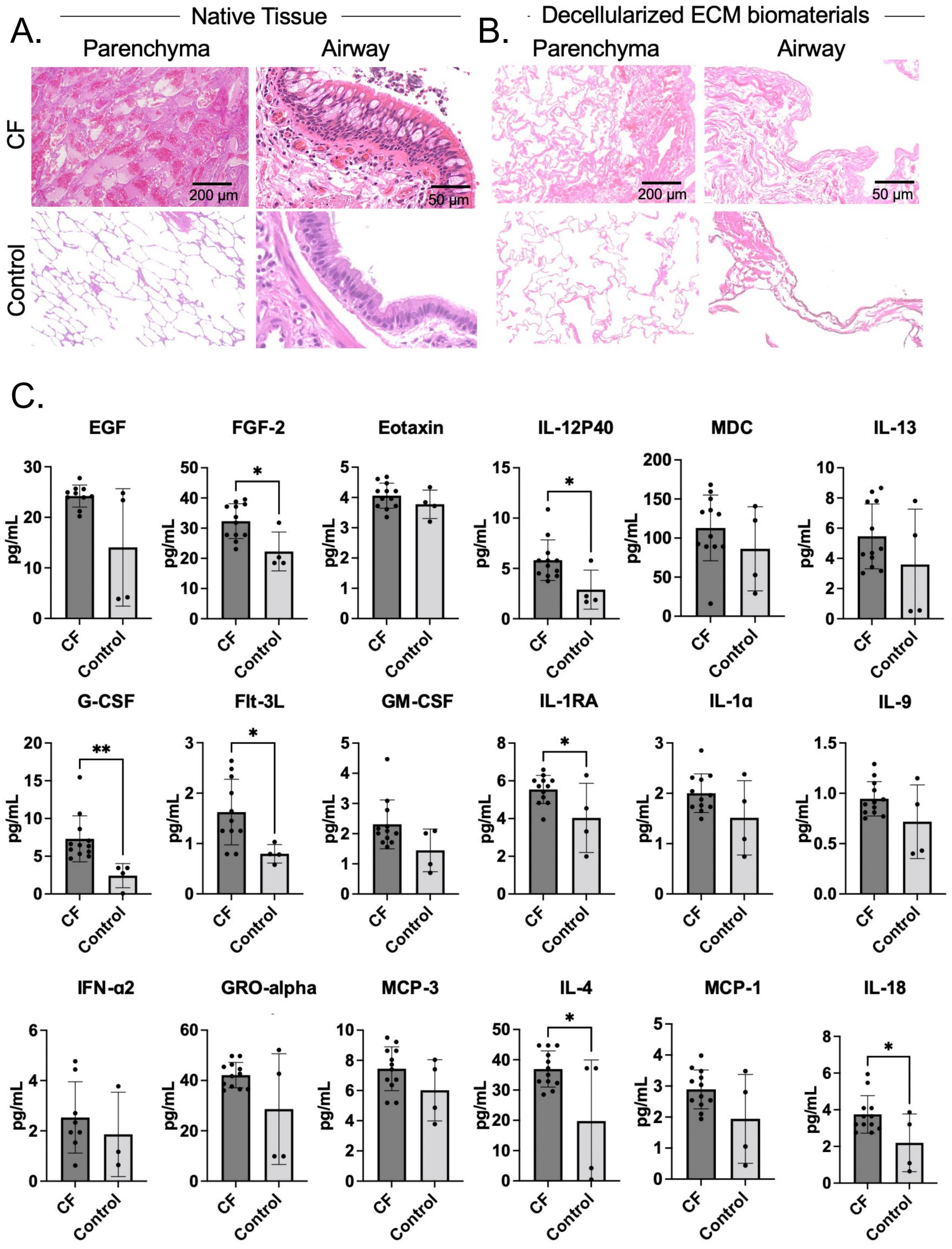

Figure S3. Comparison of CF and normal lung extracellular matrix biomaterials. Hematoxylin and eosin (H&E) of airways and parenchyma from (A) Native lung tissue and (B) decellularized extracellular matrix biomaterials. CF ECM biomaterial retains features of native CF lung including parenchymal and basement membrane derangement compared to control ECM biomaterial; (C) Cytokine levels were measured in CF and control lung ECM. EGF, epidermal growth factor; FGF-2, fibroblast growth factor-2; IL-12P40, interleukin 12B; MDC, C-C motif chemokine ligand 22; IL-13, interleukin-13; G-CSF, colony stimulating factor 3; Flt-3L, fms related receptor tyrosine kinase 3; GM-CSF, granulocyte-macrophage colony stimulating factor; IL-1RA, interleukin-1 receptor agonist; IL-1α, interleukin-1alpha; IL-9, interleukin-9; IFN-α2, interferon alpha-2; GRO alpha, C-X-C motif chemokine ligand 1; MCP-3, chemokine ligand 7; IL-4, interleukin-4; MCP-1, monocyte chemoattractant protein-1; IL-18, interleukin 18. *$p < 0.05$, **$p < 0.01$ using a student's unpaired t-test.

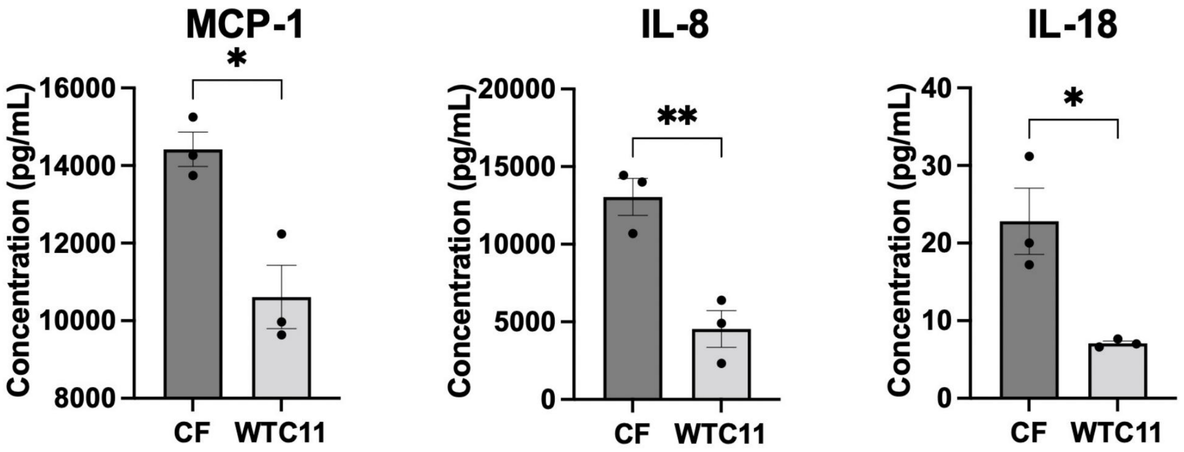

Figure S4. WTC11 and CF macrophage cytokine expression in response to CF and control lung extracellular matrix. WTC11 and CF macrophage cytokine expression at baseline. MCP-1, monocyte chemoattractant protein-1; IL-8, interleukin 8; IL-18, interleukin 18. *$p < 0.05$; **$p < 0.01$ using a student's unpaired t-test.
